# Ischemia with no obstructed coronary arteries and microvascular testing procedures: a review of utility, pharmacotherapy, and current challenges

**DOI:** 10.3389/fcvm.2025.1523352

**Published:** 2025-02-18

**Authors:** Mohammad Al Bitar, Ronney Shantouf, Ashraf Al Azzoni, Wael Al Mahmeed, Bassam Atallah

**Affiliations:** ^1^ School of Medicine, Royal College of Surgeons in Ireland, Busaiteen, Ireland; ^2^ Cleveland Clinic Abu Dhabi, Abu Dhabi, United Arab Emirates

**Keywords:** INOCA, ANOCA, microvascular resistance, microvascular dysfunction, CMD, functional testing, myocardial bridge (MB), radial artery spasm

## Abstract

Ischemia with no obstructive coronary arteries (INOCA) is an increasingly recognized condition in patients presenting with angina and positive stress tests but without significant coronary artery stenosis. This review addresses the pathophysiology, diagnostic approaches, and management strategies associated with INOCA, emphasizing epicardial coronary spasms and coronary microvascular dysfunction (CMD) as underlying mechanisms and myocardial bridging (MB) as a risk factor. Diagnostic modalities include both non-invasive techniques and invasive procedures, such as acetylcholine provocation testing, to differentiate vasospasm from microvascular causes. The paper discusses a potential interference between vasodilators used in trans-radial access and coronary spasm testing. Long-term management approaches for INOCA patients, including pharmacologic therapies and lifestyle interventions, are reviewed.

## 1 Introduction

Patients with coronary artery disease (CAD) experience several signs and symptoms, including episodes of angina and shortness of breath. Obstructive CAD is well documented and can be readily detected by conventional coronary angiograms. Patients presenting with angina with a positive stress test who end up having a left heart catheterization showing normal coronary arteries or stenosis of <50% of the artery's diameter are increasing in number (1). This presentation of anginal symptoms without hemodynamically significant stenotic lesions is termed angina with non-obstructed coronary arteries (ANOCA), which is suggestive of ischemia with non-obstructive coronary arteries (INOCA), but can also occur independently due to a lack of clear evidence of ischemia or inadequate testing (2). Myocardial infarction with non-obstructive coronary arteries (MINOCA) shows myocardial necrosis and is characterized by elevated troponin without obstructive CAD or any other non-coronary etiology. Several risk factors contribute to INOCA, including but not limited to the traditional CAD risk factors and the presence of myocardial bridging (MB).

Functional assessment of coronary arteries using invasive or non-invasive methods is needed to detect the underlying pathology of INOCA. Commonly performed non-invasive diagnostic modalities are transthoracic Doppler echocardiography (TTDE), cardiac positron emission tomography (PET), and cardiac magnetic resonance (CMR). However, these modalities often do not offer a complete comprehensive assessment given the limitations of evaluating coronary spasms. Invasive approaches involve guidewire-based assessment of the coronary circulation allowing for a more comprehensive evaluation. The invasive functional study assesses the coronary blood flow during resting and hyperemic states induced by pharmacologic agents such as adenosine or papaverine. In addition, spasm provocation testing using acetylcholine can be performed for diagnosing vasospasms. Several indices, including the fractional flow reserve (FFR), coronary flow reserve (CFR), and index of microvascular resistance (IMR), are indicative of the coronary circulation function and are obtained from different functional tests.

In this paper, we review the underlying pathologies, clinical presentation, prevalence, and risk factors for ANOCA and/or INOCA. Among different risk factors, we focus on myocardial bridging and its association with angina. Functional assessment of the coronary arteries is discussed along with a potential limitation of invasive testing through trans-radial access (TRA), which is radial artery spasms (RAS). RAS can be minimized by the administration of anti-spasmolytic vasodilators. However, this raises a concern about an interaction between the different vasodilators utilized for RAS prevention and acetylcholine administration during coronary spasm provocation testing, potentially masking spasms and reducing the sensitivity of the test. We discuss the efficacy of different vasodilating agents for RAS prevention and a reasonable approach to minimize this interaction by using vasodilators with short half-lives. Long-term management of INOCA patients comprising pharmacological disease-modifying or symptomatic therapies and non-pharmacological treatments is also reviewed.

## 2 Overview of ischemia with no obstructed coronary arteries (INOCA)

The most common underlying cause of INOCA is dysfunction of the coronary vasculature, which mainly manifests as epicardial coronary spasms, coronary microvascular dysfunction (CMD), or both combined (3). Ischemia caused by CMD presents clinically as microvascular angina (MVA), previously called cardiac syndrome X, and ischemia due to epicardial coronary spasms presents as vasospastic angina (VSA), caused by hyperconstriction of large coronary arteries with vasoconstrictor stimuli (4). The presence of INOCA in patients presenting with ANOCA can be readily detected by ECG stress testing with high specificity (5).

**Figure 1:**
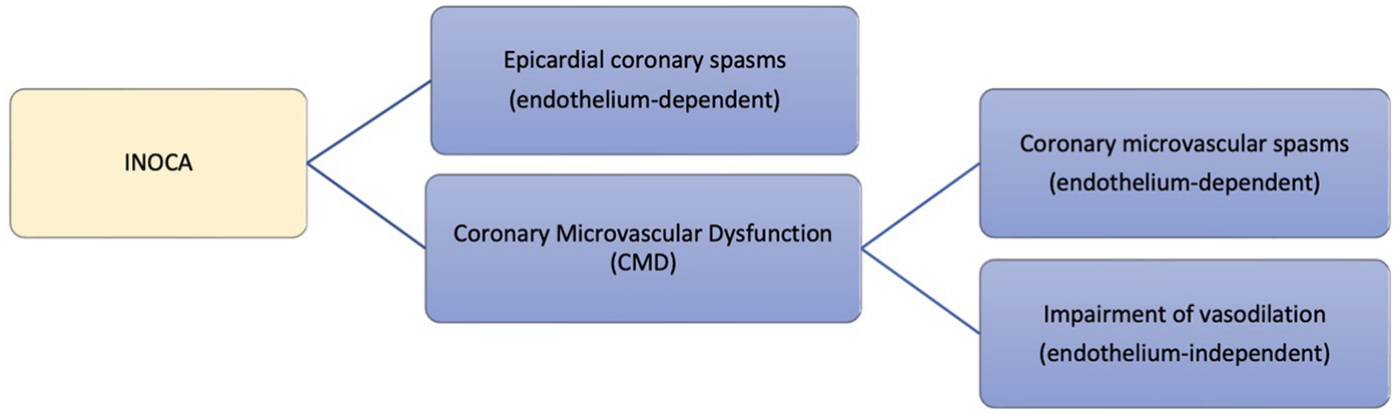
Underlying pathologies of INOCA.

As shown in Figure 1, CMD traditionally encompasses coronary microvascular spasms or vasodilation impairment and is comprised of two basic endotypes. These are endothelium-dependent dysfunction or endothelium-independent dysfunction. The endothelium-independent pathway is tested using adenosine while the endothelium-dependent pathway is evaluated via administration of acetylcholine. Other descriptors of microvascular dysfunction include structural and functional dysfunction. Structural dysfunction is thought to be caused by inward remodeling of coronary arterioles or capillary rarefaction (6). This reduces vasodilation capacity in coronary microcirculation and increases the sensitivity to vasoconstrictor stimuli (7). Functional dysfunction might be caused by the presence of endothelium dysfunction impairing vasodilation and causing vasoconstriction along the coronary circulation (8). CMD increases mortality by fourfold and increases major adverse cardiovascular events (MACE) by fivefold as demonstrated in a systematic review assessing the association of CMD and outcomes (9). CMD was found to have a role across different cardiovascular diseases other than non-obstructive CAD, including Takotsubo syndrome, angina post-PCI or CABG, obstructive CAD, heart failure, diabetic cardiomyopathy, aortic stenosis, and infiltrative heart disease (10). Moreover, a prospective study on 56 patients comparing CMD in heart failure with preserved or reduced ejection fraction (HFpEF or HFrEF) demonstrated different characteristics of CMD in both subtypes with a lower rate of recovery at follow-up in HFrEF, represented by reduced left ventricular reverse remodeling (11). These findings propose a prognostic and pathophysiological role of CMD across different cardiovascular diseases (10, 11).

According to a systematic review and meta-analysis that included 56 studies (12) investigating the provenance of CMD and vasospastic angina in patients without obstructive coronary arteries, the prevalence of CMD, coronary spasm, or a combination of both was reported as 0.41, 0.49, and 0.23, respectively. Therefore, approximately half of the population presenting without obstructive coronary arteries will have either CMD or coronary spasms. Furthermore, the prevalence of INOCA varies between different ethnicities. According to a study (13), comparing the prevalence of coronary vasomotion disorders between Japanese and Caucasian populations, CMD was more common among Caucasians, and epicardial spasms were significantly higher in Japanese patients. Another study (14) involving different ethnic groups with heart failure demonstrated that South Asians had significantly lower endothelium-mediated microvascular response to acetylcholine than Caucasians and African Caribbeans. The prevalence of non-obstructive CAD and INOCA among Middle Eastern patients is poorly investigated, and further studies are required in this region.

An established risk factor for INOCA is female gender. The Woman Ischemia Syndrome Evaluation (WISE) study recruited women with angina or suspected myocardial infraction undergoing a clinically indicated invasive coronary angiogram and other functional tests if required to diagnose ischemic heart disease. The study indicated that approximately two-thirds of the recruited women were found to have non-obstructive CAD (15, 16). Traditional risk factors contribute to INOCA, but this relationship is not well established. Also, risk factors vary for different underlying pathologies of INOCA. Older age, cigarette smoking, obesity, dyslipidemia, and hypertension are positively associated with INOCA (3, 17). Another risk factor for developing INOCA, and specifically coronary spasm, which is not well investigated is myocardial bridging (MB) (18).

## 3 Myocardial bridging and INOCA

Myocardial bridging (MB) occurs when coronary arteries run through the myocardium rather than their normal surface position (19). MB has been thought of as a benign condition as it appears harmless in most patients (20). Since coronary arteries fill during diastole, the compression of a tunneled artery during systole, known as the “milking effect,” should not affect myocardial perfusion (21). However, cardiovascular complications have been reported in some cases of MB, including vasospastic angina and acute myocardial infarctions. Moreover, several studies found a significant association between MB and myocardial ischemia (18).

Intravascular ultrasounds assessing MB demonstrated delayed compression release of the affected artery until early diastole, which can potentially precipitate ischemia (22). Persistent lumen diameter reduction during mid-diastole was also reported (23). Consequently, conditions that shorten the diastolic time, such as tachycardia, will contribute to ischemia. Another underlying mechanism of ischemia in MB is endothelial dysfunction due to systolic kinking of arteries, causing less production of vasoactive substances like nitric oxide (NO) and endothelin (24). Vascular dysfunction caused by MB contributes to the occurrence of vasospasms (25). Atherosclerosis proximally to a tunneled artery has been reported as a complication to MB due to decreased shear stress and increased vasoactive substances in that segment (24).

Variable prevalence of MB was reported among different studies and based on the diagnostic modality. A 2017 meta-analysis reported an overall 19% prevalence of MB (26). However, it was reported as high as 42% on autopsy despite a 6% prevalence of coronary angiography. Furthermore, computed tomography studies revealed a prevalence of 22% (26). Myocardial bridging contributes to INOCA through several mechanisms. A retrospective study of 62 patients with INOCA reported the presence of MB in 15 patients (24%). Coronary spasms were more common among patients with MB. However, coronary microvascular function was similar in both patient groups. This study demonstrates that MB predisposes patients with INOCA to coronary spasms but has a limited effect on coronary microvascular dysfunction (CMD) (27).

MB was associated with increased occurrence of coronary spasm and MINOCA in a prospective study on 310 participants with stable non-obstructive CAD or MINOCA. The percentage of patients with a positive acetylcholine test was 59% (183 patients), and compared with patients with a negative test, a higher prevalence of MINOCA (53.6% vs 33.9%) and MB was reported (23% vs 8.7%). Moreover, the overall prevalence of MB among the study participants was 17.1%, who presented with a higher rate of major adverse cardiac events (MACE) and decreased Seattle Angina Questionnaire scores compared with patients without MB. Overall, coexistent MB and positive acetylcholine test reported the poorest clinical outcome (28). However, more studies are needed to further investigate the relationship between INOCA and MB.

## 4 Non-invasive and invasive testing for INOCA

Conventional angiography or coronary computed tomographic angiography (CCTA) demonstrating non-obstructive coronary artery disease in a patient with INOCA/ANOCA necessitates further evaluation through invasive or non-invasive functional assessment for CMD. Despite the advantages of non-invasive testing, such as fewer procedural complications, an invasive approach can identify vasospasm using provocation testing, which is not clinically feasible through non-invasive methods (29). The CorMicA randomized controlled trial showed that tailored stratified medical therapy through interventional diagnostic procedures or functional tests in patients with ANOCA improved patient outcomes (30).

The most common approach for invasively assessing hemodynamics is through a coronary wire equipped with pressure and temperature sensors allowing for complete coronary physiologic assessment such as the PressureWire X Guidewire by Abbott Laboratories (31). The combination of a pressure and temperature sensor allows for both pressure changes and flow characteristics. This approach relies on thermodilution by either bolus or continuous infusions of saline to assess the coronary microvasculature. Continuous thermodilution is a novel method that directly measures absolute coronary blood flow and microvascular resistance (32). Compared to bolus thermodilution, continuous thermodilution was found more precise and demonstrated a significant decrease in variability (33, 34). Another approach includes Doppler-wire assessment measuring Doppler velocity coronary flow (35).

**Figure 2:**
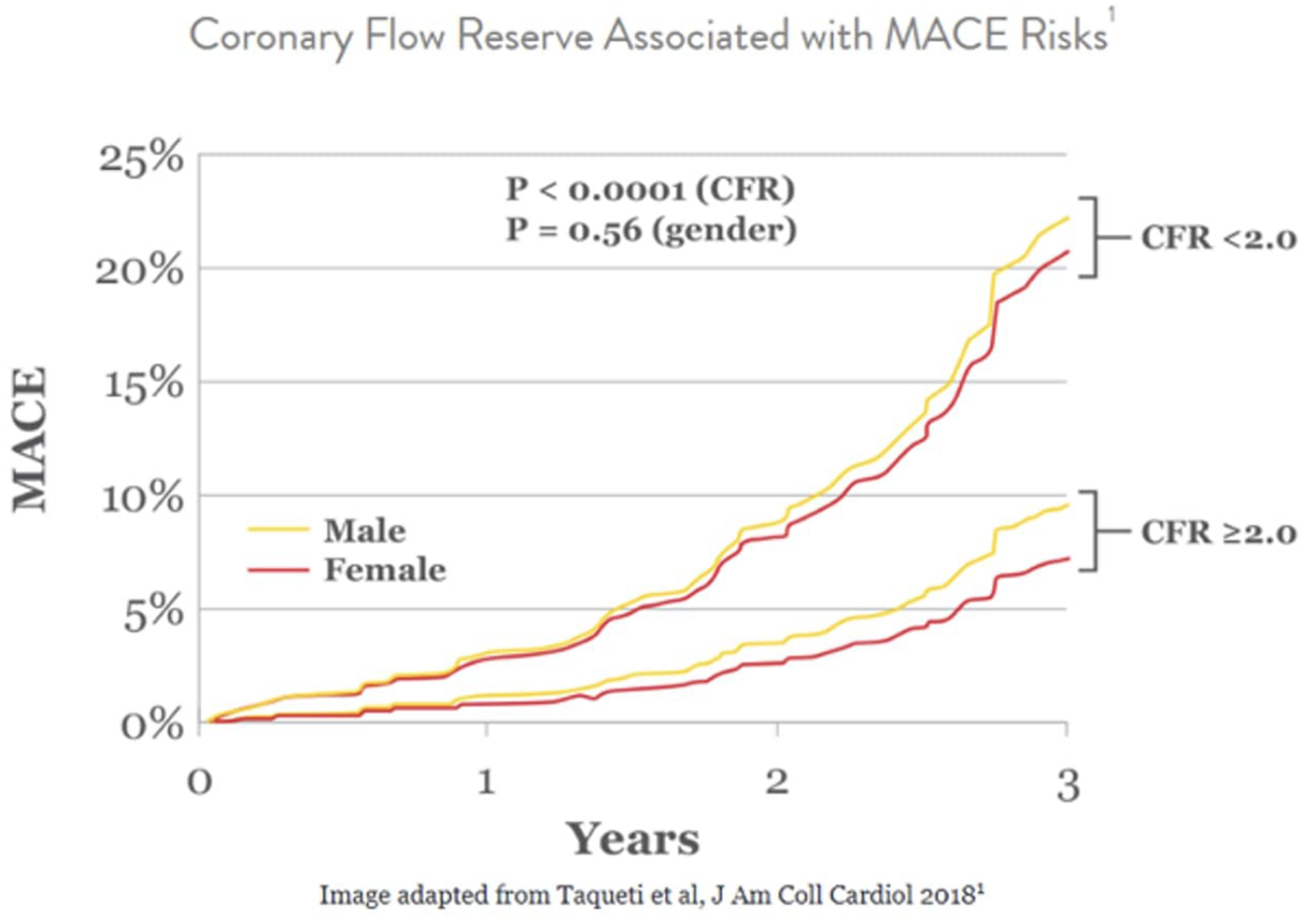
Relationship between CFR and incidence of MACE among males and females.

The most studied index and gold standard for evaluating stenotic epicardial arteries is the fractional flow reserve (FFR) (36). FFR is defined as the ratio of pressure distal and proximal to stenosis measured by the ratio of pressure distal to the lesion and the pressure in the aorta under maximal hyperemia (37). A ratio of >0.80 rules out hemodynamically and functionally significant stenosis (38). However, FFR alone does not assess the microcirculation. Subsequently, the coronary flow reserve (CFR) and the index of microvascular resistance (IMR) are used. The administration of a hyperemia-inducing agent such as adenosine is required to measure CFR. Alternatively, papaverine or regadenoson can be used. This allows for the evaluation of the endothelium-independent pathway. CFR is a measure of blood flow increase that the coronary circulation can yield in response to stress (39). It reflects the vasodilator capacity of both the epicardial and microvascular circulation with an abnormal value defined as <2–2.5 (40). When hemodynamically significant stenosis of the epicardial vessel is ruled out, CFR can then serve as an indicator of the microcirculation's ability to augment flow under hyperemic states. CFR, reflecting CMD, and major adverse cardiovascular events (MACE) are inversely related, as MACE occurred more frequently and earlier with lower CFR values as shown in Figure 2 (41). IMR, which is specific to the microcirculation with an abnormal value of >25, is calculated by measuring distal coronary pressure at maximal hyperemia and the average period of time that blood spends in a vessel (39). The microvascular resistance reserve (MRR) is a novel index specific to the coronary microvasculature, operator-independent, and unaffected by coronary autoregulation, epicardial resistance, or myocardial mass (42). MRR is defined as the microvascular resistance at rest when the epicardial artery is normally divided by microvascular resistance under maximal hyperemia and can be measured by dividing CFR by FFR when obtained through continuous thermodilution (42). Furthermore, unlike CFR, MRR is independent of epicardial resistance as CFR was shown to decrease in response to increasing epicardial resistance separating from MRR (42). Other than continuous thermodilution, MRR can also be obtained through several modalities including bolus thermodilution, Doppler velocity, or non-invasively through CT, MRI, or PET (33, 42). However, continuous thermodilution has shown superiority in precision when compared with bolus thermodilution, and does not require a hyperemia-inducing agent (33, 43). The ILIAS study on the prognosis of patients without obstructive coronary artery disease and with impaired CFR and microvascular resistance values demonstrated an increase in major adverse cardiac events (MACE) and target vessel failure (TVF) at 5 years with impaired CFR, but not with abnormal microvascular resistance (44). This demonstrates the poor prognostic value of microvascular resistance compared with CFR.

Complete functional evaluation can proceed to assess the endothelium-dependent microvascular pathway through provocation testing most commonly using intracoronary acetylcholine or ergonovine following a fixed protocol. Angina with a reduction of >90% in the diameter of epicardial vessels and ECG abnormalities indicate epicardial vasospastic angina (45, 46). While still being heavily researched, the accepted definition of microvascular angina includes the induction of angina and ischemic ECG changes without a >90% reduction of the epicardial arteries. This would indicate endothelium-dependent microvascular spasm (47). Coexistence of both epicardial and microvascular spasms occurs with undetermined frequency due to its identification difficulty (48). A novel method to detect coexistent microvascular and epicardial spasms is the acetylcholine rechallenge test, where coronary arteries are rechallenged with acetylcholine after administering an anti-vasospastic agent such as nitroglycerin. A study (48) on patients with epicardial spasms demonstrated that 48% had coexisting microvascular spasms. In addition, microvascular spasms were diagnosed at lower doses of acetylcholine, while epicardial spasms were detected with higher doses (48). Of note, acetylcholine might cause vasoconstriction, affecting adenosine studies by disrupting the resting hemodynamic state. Hence, starting with adenosine testing might be a better approach.

Several non-invasive modalities can be utilized to diagnose INOCA/ANOCA. Transthoracic doppler echocardiography (TTDE) can non-invasively measure CFR by measuring flow velocity in the LAD artery to identify CMD (49–51). However, it poorly differentiates epicardial from microvascular dysfunction and is affected by intra-operator variability (46). Positron emission tomography (PET) is the most reliable and widely preferred modality for the evaluation of microvascular disease (52). PET measures MBF and myocardial flow reserve (MFR), which is obtained from MBF values at rest and hyperemia. An MFR value of <1.5 suggests microvascular disease (53). MBF can be also evaluated through cardiac MRI to assess for microvascular dysfunction. The myocardial perfusion reserve (MPR) index can be obtained from myocardial perfusion at rest and hyperemia with an abnormal value of <1.5 (54). ECG stress testing conventionally represents obstructive coronary but has shown accurate prediction of non-obstructive coronary artery disease in recent studies. Stress ECG was found to be 100% specific for identifying ischemia underlined by CMD in patients with ANOCA (5). However, functional assessment invasively remains superior to non-invasive stress ECG to identify microvascular disease as shown to significantly decrease the false discovery rate in the UZ clear study (55).

In line with the 2024 ESC guidelines (46), Class 1 recommendations state that invasive functional testing through a coronary guidewire to obtain CFR and IMR should be considered to assess patients presenting with coronary microvascular angina and persistent symptoms without stenosis or a change in FFR/iFR. Conversely, non-invasive testing for INOCA through modalities, such as transthoracic Doppler or stress echocardiograph, cardiac MRI, and PET to assess CFR, was listed as a Class 2b recommendation. Additionally, Class 1 recommendations for the diagnosis of vasospastic angina indicate invasive assessment with an intracoronary provocation test to identify spasms in the absence of obstruction on angiography.

## 5 Prevention of radial artery spasm and possible impact on invasive coronary spasm assessment

Trans-radial access (TRA) has several advantages over trans-femoral access (TFA), including decreased hemorrhage risk, shorter recovery time, higher patient satisfaction, and decreased procedural costs (56). However, a major limiting factor to TRA is the risk of developing radial artery spasms (RAS) and thus the administration of anti-spasmodic medications or the use of hydrophilic long radial sheaths (57, 58) is commonly practiced to overcome RAS. Common spasmolytic agents used during trans-radial access are nitroglycerin, verapamil, or a combination of vasodilators in a cocktail, mostly including nitroglycerin. Other used agents are demonstrated in Table 1. The optimal agent is debated, and the preferred choice varies in clinical practice (59). It is unknown whether or not using vasodilators during radial access potentially impacts vasoreactivity results due to the pre-administration of anti-spasmodic medications in the radial artery prior to intracoronary acetylcholine administration. Given the short procedural time and the opposed effects of acetylcholine and vasodilators, using agents with short half-lives (or no anti-spasmatic agents at all) into the radial artery to limit the occurrence of any interaction would seem prudent. Alternatively, the use of hydrophilic long radial sheaths has shown efficacy in overcoming RAS (57, 58). Nitroglycerin has the shortest half-life compared with other vasodilators used in this setting such as verapamil (3 min vs 3–5 h), thus offering the advantage of less potential for overlapping with the desired acetylcholine effect (60, 61). Given the half-life of nitroglycerin, we recommend waiting at least 10 min prior to the initiation of vasoreactivity testing from the last dose of intra-arterial nitroglycerin administration to ensure multiple half-live have passed. This also applies if FFR and/or CFR testing is to be performed prior to acetylcholine testing as typically intracoronary nitroglycerin is administered prior to FFR and/or CFR testing.

**Table 1:** Half-lives, mechanisms of action, and studies assessing different agents for the prevention of radial artery spasms (RAS).

Agent	Half-life	Mechanism of action	Study title	Authors (year)	Study design	Intervention	Main results
Verapamil	3–5 h (60)	Non-dihydropyridine calcium channel blocker	“How to limit radial artery spasm during percutaneous coronary interventions: the spasmolytic agents to avoid spasm during transradial percutaneous coronary interventions (SPASM3) study” (62)	Rosencher et al. (2014)	Randomized controlled trial	Verapamil or ISDN or diltiazem	- RAS prevalence was 20.1% in the whole population which was reduced significantly by verapamil and ISDN compared with diltiazem (16.2, 17.2, and 26.6%, respectively. *P* < 0.006) - Patients treated with verapamil compared with ISDN and diltiazem had less severe pain (1.3%, 2.8%, and 2.9%) (*P* = 0.43) and less severe RAS (5.1%, 6.2%, and 9.5%, respectively) (*P* = 0.13)
Diltiazem	2–5 h (63)	Non-dihydropyridine calcium channel blocker					
Isosorbide dinitrate (ISDN)	1–2 h (64)	Long-acting nitrate, stimulates soluble guanylate cyclase (GC), which results in increased cyclic GMP (cGMP) levels (65)					
Nitroglycerin	3 min (61)	Short-acting nitrate, stimulates soluble guanylate cyclase (GC), which results in increased cyclic GMP (cGMP) levels (65)	“A simple and effective regimen for prevention of radial artery spasm during coronary catheterization” (66)	Chen et al. (2005)	Randomized controlled trial	Heparin, nitroglycerin, and verapamil (Group A) or heparin and nitroglycerin (Group B) or heparin alone (Group C)	- RAS experienced by 3.8% of patients in Group A, 4.4% in B, and 20.4% in C - - Groups A and B were similar in RAS occurrence (*P* = 0.804). However, Group C showed a significantly higher occurrence of RAS
Phentolamine	19 min (67)	Nonselective alpha-blocker	“Assessment of the efficacy of phentolamine to prevent radial artery spasm during cardiac catheterization procedures: a randomized study comparing phentolamine vs. verapamil” (68)	Ruiz-Salmerón et al. (2005)	Randomized double-blind controlled trial	Phentolamine or verapamil	- Significant radial artery diameter increase was observed with both vasodilators - Verapamil superior to phentolamine in the prevention of RAS (13.2% and 23.2%, respectively) (*P* = 0.004) - Radial occlusion rate was similar in both groups
Nitroprusside	1–2 min (69)	Increases production of nitric oxide (NO)	“Nitroglycerin, nitroprusside, or both, in preventing radial artery spasm during transradial artery catheterization” (70)	Coppola et al. (2006)	Randomized controlled trial	Nitroglycerin (Group A) or nitroprusside (Group B) or nitroglycerin and nitroprusside (Group C) All groups also received intra-arterial heparin, lidocaine, and diltiazem	- 44 out of 379 patients (11.6%) experienced RAS, with similar occurrence in all groups (*P* = 0.597)
Nicorandil	1 h (71)	Activation of potassium channel and a nitrate-like effect (72)	“Comparative study of nicorandil and a spasmolytic cocktail in preventing radial artery spasm during transradial coronary angiography” (73)	Kim et al. (2007)	Randomized controlled trial	Nicorandil or cocktail of normal saline and verapamil	- Both agents significantly dilated the radial artery when administered trans-radially (*P* < 0.001) - Nicorandil significantly increased of the radial artery mean diameter compared with the cocktail at mid-segment - No statistically significant occurrence difference of RAS
Molsidomine	1 h (74)	Acts through its metabolite SIN-1 to produce nitric oxide (NO) (75)	“Prevention of arterial spasm during percutaneous coronary interventions through radial artery: the SPASM study” (76)	Varenne et al. (2006)	Randomized controlled trial	Placebo or molsidomine 1 mg or verapamil 2.5 mg or verapamil 5 mg or verapamil 2.5 mg and molsidomine 1 mg	- RAS occurrence reduced in all groups but was lowest with verapamil and molsidomine (4.9%), compared with verapamil 2.5 mg (8.3%), verapamil 5 mg (7.9%), molsidomine 1 mg (13.3%), and a placebo (22.2%) (*P* < 0.0001)
Magnesium sulphate	5 h (77)	Mainly a calcium antagonist	“Magnesium sulphate during transradial cardiac catheterization: a new use for an old drug?” (78)	Byrne et al. (2008)	Prospective, double-blind, randomized trial	Magnesium sulfate or verapamil	- Both agents increased radial diameter (*P* < 0.01) with greater effect with magnesium (*P* < 0.05) - Mean arterial pressure (MAP) was decreased on verapamil but no change observed in magnesium

## 6 Long-term management of ANOCA and/or INOCA

Pharmacological therapeutic approaches for ANOCA and/or INOCA include disease-modifying therapies or symptomatic treatments. Non-pharmacological management is also essential and involves lifestyle modifications and risk factor management.

Disease-modifying therapies include statins, angiotensin-converting enzyme (ACE) inhibitors, and low-dose aspirin. Statins can potentially enhance endothelial function and reduce blood viscosity and shear stress, improving CFR and the microcirculation's function (79). A study (80) on 68 patients with microvascular angina, randomized into three groups receiving fluvastatin, diltiazem, or a combination of both, demonstrated improved CFR in all groups after 90 days. However, a combination of fluvastatin and diltiazem was the most effective (80). The beneficial effect of these drugs might be attributed to increasing nitric oxide and reducing endothelin-1 (80). ACE inhibitors significantly improve CFR, exercise tolerance, and angina symptoms (81, 82). A randomized placebo-controlled trial on 45 patients with ANOCA evaluated the effect of a combination of statins and ACE inhibitors on the antioxidant enzyme superoxide dismutase (SOD), reflecting oxidative stress, and on exercise capacity and Seattle Angina Questionnaire (SAQ) scores, reflecting quality of life. At 6 months of treatment, the intervention group receiving atorvastatin and ramipril showed significant reduction (*P* = 0.001) in SOD compared with placebo. In addition, increased exercise duration by 23.46% and SAQ score by 64.1% were observed with the treatment, indicating improved quality of life (82).

Anti-anginal treatments vary for different underlying mechanisms of INOCA, so tailored treatment plans are required as demonstrated in the CorMicA study where stratified medical therapy improved outcomes (83). The first-line treatment for patients with microvascular angina is beta-blockers, but they are contraindicated for coronary vasospasm (84). In patients with coronary vasospasm, calcium channel blockers (CCB) are considered first-line therapy and highly effective, as demonstrated in a double-blind placebo-controlled study, where 21 patients with angina at rest were randomized to a placebo, isosorbide-5-mononitrate, or nifedipine (85). Results showed that both drugs significantly reduced spontaneous and induced ischemic attacks (85). Both dihydropyridine and non-dihydropyridine calcium channel blockers have a well-established efficacy in relieving angina as demonstrated in many studies. However, choosing an agent has to be patient-tailored taking factors such as specific adverse reactions to each drug into consideration (86). In a meta-analysis of four studies on Japanese vasospastic angina patients, 1,349 patients who tested positive on the coronary spasm provocation test received one of four CCBs, which are benidipine, amlodipine, nifedipine, and diltiazem. Patients treated with benidipine showed significantly fewer major adverse cardiovascular events (MACE), which demonstrated a more beneficial prognostic effect of benidipine compared with amlodipine, nifedipine, and diltiazem (87). Furthermore, the EDIT-CMD randomized clinical trial (88) assessed the efficacy of diltiazem compared with placebo in patients identified with either vasospasms or microvascular dysfunction on coronary function testing. On a second coronary function test 6 weeks after treatment, coronary vasomotor dysfunction, symptoms, and quality of life were similar between the patients treated with diltiazem and placebo. However, epicardial spams were significantly reduced with diltiazem. CCBs are also effective for CMD, thus in cases where the underlying mechanism of INOCA is unknown or treatment with beta-blockers for CMD is not sufficient, CCBs are recommended. Long-acting nitrates can be considered second-line therapy for vasospastic angina but are ineffective for microvascular angina with the potential to aggravate the symptoms (89–91).

Nicorandil acts as a potassium channel activator and a nitrate-like factor with established efficacy for both CMD and coronary spasms and can be used as a third-line treatment for both endotypes (92–94). A randomized controlled trial on 13 patients with microvascular angina showed that nicorandil improved exercise-induced myocardial ischemia without modification of cardiac autonomic activity, which demonstrates a direct vasodilatory effect of nicorandil on the microcirculation (94). Another study assessing the effect of nicorandil on 10 patients with spontaneous and ergonovine-evoked coronary spasms showed complete spasm relief in all patients (92).

Studies demonstrated conflicting results on the efficacy of ranolazine for the treatment of CMD. A randomized controlled crossover trial on 20 females with ANOCA and ischemia treated with ranolazine demonstrated higher Seattle Angina Questionnaire (SAQ) scores and improved myocardial ischemia among women with low CFR (95). Moreover, ranolazine significantly increased CFR in a double-blind, placebo-controlled trial on 58 patients with angina and INOCA (96). Several studies showed improved outcomes with the use of ranolazine (95–97). However, it was found generally ineffective in one trial (98).

The ChaMP-CMD randomized controlled study (99) assessed the effect of amlodipine and ranolazine on treadmill exercise time and Seattle Angina Questionnaire summary score in patients with CMD defined as CFR <2.5 compared with patients with CFR >2.5. Patients with CMD showed prolonged treadmill exercise time on both drugs compared with the control group. However, the Seattle Angina Questionnaire score was improved in CMD patients taking ranolazine but not in response to amlodipine. The study suggested that only patients with decreased CFR might respond to anti-ischemic therapy emphasizing the importance of stratified medical therapy through measurement of CFR.

**Figure 3:**
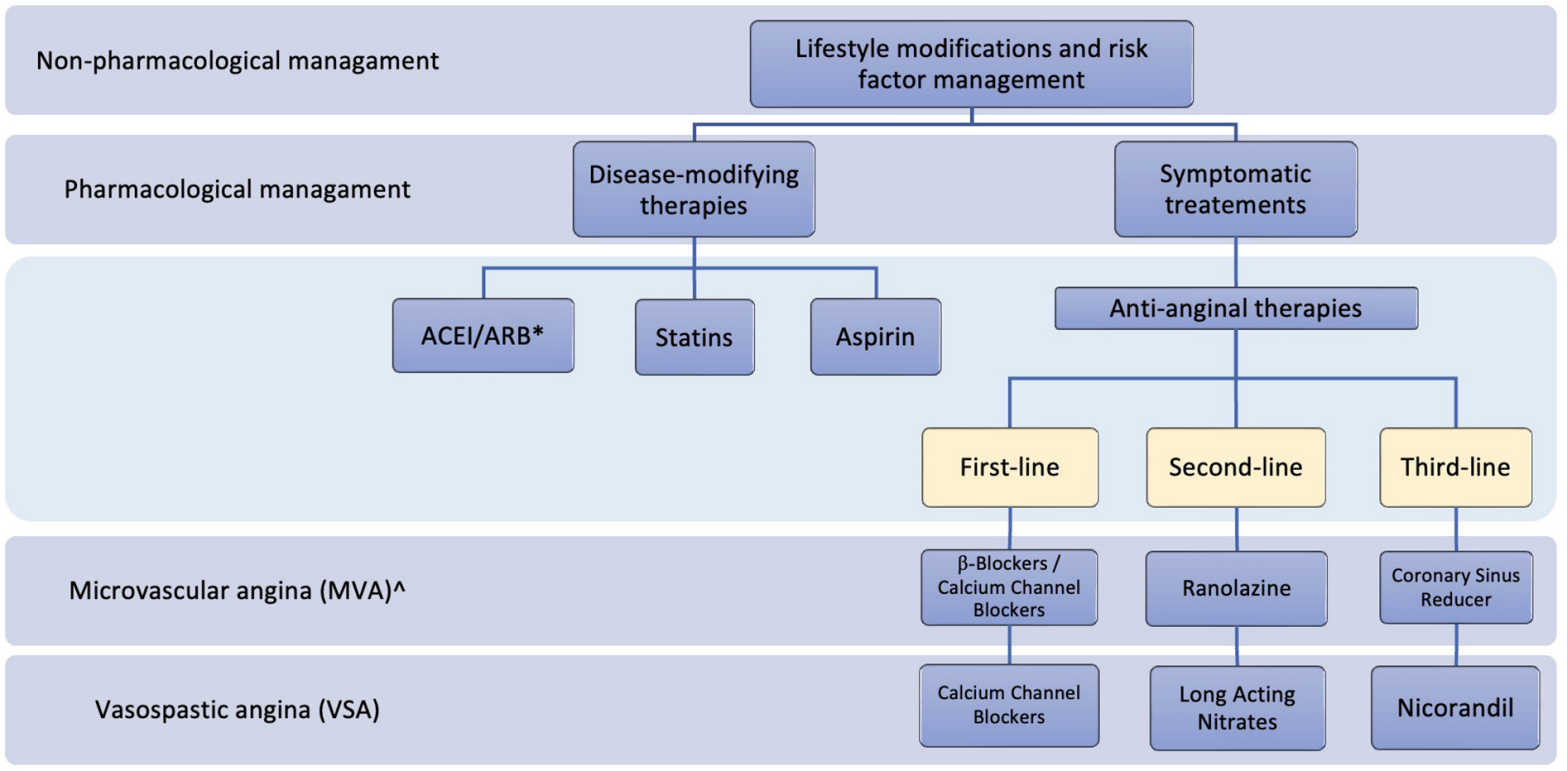
Summary of the management plan for patients with INOCA. *ACEI, Angiotensin-Converting Enzyme Inhibitors; ARB, angiotensin receptor blockers; ^, endothelium-independent.

Other treatments can be used alternatively or in combination with the therapies mentioned above and demonstrated their efficacy in some studies. These include imipramine (100), ivabradine (97), aminophylline (101), L-arginine (102), phosphodiesterase (PDE) inhibitors (103), metformin (104), trimetazidine (105), and Rho-kinase inhibitors (106). However, the benefits of these therapies are not well established. The mechanisms of action and efficacy studies for the different agents used for the long-term management of INOCA are discussed in Table 2. Figure 3 summarizes the management plan for patients with INOCA of different underlying pathologies.

**Table 2:** Mechanisms of action and studies assessing different agents for the long-term management of INOCA.

Agent	Mechanism of action	Study title	Authors (year)	Study design	Intervention	Main results
Disease-modifying therapies						
Statins	Lipid-lowering effects, enhancement of endothelial function and wall shear stress, and reduction in blood viscosity (79)	“Moderate-dose atorvastatin improves arterial endothelial function in patients with angina pectoris and normal coronary angiogram: a pilot study” (112)	Kabaklić et al. (2017)	Pilot, double-blind, placebo-controlled, randomized intervention study	- 58 patients with ANOCA/INOCA randomized to 20 mg of atorvastatin OD (Group A) or placebo (group P) for 6 months	- Unlike the placebo group, patients on statins showed improvement in flow-mediated dilation of the brachial artery (*P* < 0.001) - Improvement in arterial stiffness in the stating group (*P* = 0.077) - SAQ scores were generally improved
		“Statin therapy is associated with lower all-cause mortality in patients with non-obstructive coronary artery disease” (113)	Hwang et al. (2015)	Retrospective cohort study	- Statins were prescribed to 1,983 out of 8,372 patients with non-obstructive CAD	- Statin therapy was associated with lower risks of all-cause mortality (*p* < 0.0001) - Statin therapy was linked to better clinical outcomes
		“Angiotensin-converting enzyme inhibitors and 3-hydroxy-3-methylglutaryl coenzyme A reductase in cardiac syndrome X” (82)	Pizzi et al. (2004)	Randomized controlled trial	- 45 participants with syndrome X randomly assigned for 6 months to ramipril and atorvastatin or placebo	- The treatment group showed a significant reduction in superoxide dismutase (decreased oxidative stress) (*P* = 0.001) and no changes with placebo - Improved QoL with the treatment was observed
Angiotensin-onverting enzyme (ACE) inhibitors	Inhibition of angiotensin 2 production exerting a vasodilatory effect (114)					
Low-dose aspirin	Antiplatelet effects through inhibition of thromboxane A2 (TXA2) production (115)	“Aspirin in the primary and secondary prevention of vascular disease: collaborative meta-analysis of individual participant data from randomized trials” (116)	Antithrombotic Trialists’ (ATT) Collaboration et al. (2009)	Meta-analysis of clinical trials	- Meta-analyses of serious vascular events and major bleeds on 22 trials that evaluated long-term aspirin compared with control	- Reduction of serious vascular events was more observed with aspirin
Symptomatic anti-anginal therapies						
Beta-blockers	Mainly blocking B1 and B2 receptors with varying specificities and effects depending on the target (117)	“Does the β-blocker nebivolol increase coronary flow reserve?” (118)	Togni et al. (2007)	Controlled trial	- Nebivolol was administered to 8 patients with CAD and 10 controls	- CFR increased in both control and intervention groups - Collateral flow index (CFI) decreased with the intervention in CAD patients - Suggests an impact of nebivolol on myocardial oxygen consumption
		“Comparison of verapamil versus propranolol therapy in syndrome X” (119)	Bugiardini et al. (1989)	Randomized double-blind crossover placebo-controlled trial	- 320 mg verapamil OD, 120–160 mg propranolol OD, or placebo were assigned to 16 patients with syndrome X for 1 week each	- Daily occurrence of ischemia was significantly less on propranolol compared with placebo (*P* < 0.0005) - Verapamil did not show any significant changes
Calcium channel blockers	Antagonists of calcium channels and classified as non-dihydropyridines and dihydropyridines depending on the targeted tissue (120)	“Efficacy of calcium channel blocker therapy for angina pectoris resulting from small-vessel coronary artery disease and abnormal vasodilator reserve” (121)	Cannon et al. (1985)	Randomized, double-blind, placebo-controlled outpatient study	- 26 participants with angina randomized to receive verapamil (17 patients) or nifedipine (9 patients) with 1 month period for each - Patients assessed with exercise tolerance test	- Participants on CCBs reported less recurrence of angina (*P* < 0.001) and required less NTG consumption (*P* < 0.001) <0.001) compared with placebo - Slight significant prolongation in exercise duration was observed during drug treatment compared with placebo (*P* < 0.025)
		“Efficacy of isosorbide-5-mononitrate versus nifedipine in preventing spontaneous and ergonovine-induced myocardial ischaemia. A double-blind, placebo-controlled study” (85)	Lombardi et al. (1993)	Double-blind randomized trial	- 21 patients with angina at rest randomized to receive placebo or either oral isosorbide-5-mononitrate or oral nifedipine	- Both nifedipine and isosorbide-5-mononitrate significantly reduced spontaneous and induced ischemic attacks
Nitrates	Stimulate soluble guanylate cyclase (GC), which results in increased cyclic GMP (cGMP) levels (65)					
Nicorandil	Activation of potassium channel and a nitrate-like effect (51)	“Effects of short-term treatment of nicorandil on exercise-induced myocardial ischemia and abnormal cardiac autonomic activity in microvascular angina” (94)	Chen et al. (1997)	Randomized, double-blind, placebo-controlled, crossover study	- 13 patients with MVA treated with 5 mg nicorandil TID or placebo - Exercise tolerance test and ECG were obtained	- Nicorandil increased duration to 1 mm ST depression (*P* = 0.026) and exercise duration (*P* = 0.036). - Decreased maximum exercise ST depression on nicorandil (*P* = 0.083) - Immediate improvement in ischemia on nicorandil
Ranolazine	Blockage of late sodium current and prevention of cytosolic calcium rise (122)	“Effects of ranolazine on noninvasive coronary flow reserve in patients with myocardial ischemia but without obstructive coronary artery disease” (96)	Tagliamonte et al. (2014)	Double-blind, placebo-controlled trial	- 500 mg ranolazine BD or placebo for 8 weeks given to 58 patients with angina and ischemia	- Ranolazine increased CFR significantly (*P* = 0.005) with no change with placebo
		“Ranolazine improves angina in women with evidence of myocardial ischemia but no obstructive coronary artery disease” (95)	Mehta et al. (2011)	Pilot randomized, double-blind, placebo-controlled, crossover trial	- 20 females with ANOCA ≥ 10% ischemia treated for 4 weeks with ranolazine - SAQ scores, CMR ± CFR, were assessed	- Significantly higher SAQ scores in the ranolazine group compared with placebo - Ranolazine increased CMR myocardial perfusion reserve index (*P* = 0.074)
Other treatments used alternatively or in combination						
Imipramine	Tricyclic antidepressant and has type 1 antiarrhythmic effects (123)	“Imipramine in patients with chest pain despite normal coronary angiograms” (100)	Cannon et al. (1994)	Randomized, double-blind, placebo-controlled trial	- Sixty patients randomized to 0.1 mg clonidine BD, 50 mg imipramine OD, or placebo.	- Significantly more reduction of chest pain occurrence in both the clonidine and imipramine groups compared with placebo
Ivabradine	Heart rate-lowering effect by inhibiting the cardiac pacemaker current (124)	“Effects of ivabradine and ranolazine in patients with microvascular angina pectoris” (97)	Villano et al. (2013)	Randomized controlled trial	- 46 participants with microvascular angina uncontrolled on anti-ischemics treated for 4 weeks with 5 mg ivabradine BD, 375 mg ranolazine BD, or placebo.	- Enhanced quality of life in the treatment groups (*P* < 0.01) - Ranolazine improved 1 mm ST-segment depression time and ST duration
Aminophylline	Adenosine receptor blockage (101)	“Effect of oral aminophylline in patients with angina and normal coronary arteriograms (cardiac syndrome X)” (101)	Elliott et al. (1997)	Randomized controlled trial	- 13 patients with syndrome X treated for 3 weeks with either aminophylline PO or placebo. - Exercise tolerance test (ETT) and ECG performed	- Aminophylline delayed angina on ETT compared with placebo (*P* = 0.004) - No significant difference in peak exercise ST depression. - Less occurrence of chest pain on aminophylline - Unchanged occurrence and duration of ST depression
L-Arginine	Substrate for nitric oxide synthase (125)	“Long-term L-arginine supplementation improves small-vessel coronary endothelial function in humans” (102)	Lerman et al. (1998)	Randomized controlled trial	- Twenty-six patients randomized to either oral l-arginine (3 g three times daily) or placebo - Endothelium-dependent CFR response to Ach measured at baseline and 6 months after therapy.	- Increased CFR in the l-arginine group after 6 months (*P* < 0.05) - Enhanced symptoms and decreased endothelin concentrations with L-arginine
Phosphodiesterase (PDE) inhibitors	Inhibition of PDE enzymes preventing cGMP or cAMP causing relaxation and vasodilatory effects in target cells (126)	“Effect of phosphodiesterase type 5 inhibition on microvascular coronary dysfunction in women: a Women's Ischemia Syndrome Evaluation (WISE) Ancillary Study” (103)	Denardo et al. (2011)	Clinical ancillary study	- 23 symptomatic females with no obstructive CAD and CFR ≤3.0 - 100 mg oral sildenafil administered and CFR measured afterwards	- Increased CFR from 2.1 to 2.7 in women with baseline CFR ≤2.5 (*P* = 0.006) - No difference in CFR in patients with a baseline of >2.5 (*P* = 0.70)
Metformin	AMP-activated protein kinase (AMPK) agonist (127)	“Effects of metformin on microvascular function and exercise tolerance in women with angina and normal coronary arteries: a randomized, double-blind, placebo-controlled study” (104)	Jadhav et al. (2006)	Randomized controlled trial	- 33 nondiabetic women with normal angiography and positive stress tests were given metformin 500 mg BD or placebo for 8 weeks	- The metformin group showed enhanced endothelium-dependent microvascular response (*P* = 0.0003). However, endothelium-dependent response was not changed - Improvement in maximal ST-segment depression (*P* = 0.013), and chest pain (*P* = 0.056) - Better Duke score (*P* = 0.008)
Trimetazidine	Inhibition of β-oxidation of free fatty acid (128)	“The effect of trimetazidine in the treatment of microvascular angina” (105)	Nalbantgil et al. (1999)	Placebo-controlled, double-blind study	- 35 patients with CMD treated with either trimetazidine (60 mg OD) or placebo - Patients were assessed by stress testing	- Exercise time and the >1 mm depression of ST-segment time were increased in the trimetazidine compared with placebo Additionally, ST depression was decreased
Rho-kinase inhibitors	Inhibition of Rho-kinase which modulates calcium sensitivity in muscle cells (129)	“Rho-kinase inhibition with intracoronary fasudil prevents myocardial ischemia in patients with coronary microvascular spasm” (106)	Mohri et al. (2003)	Clinical trial	- 18 patients with ANOCA and Ach testing inducing ischemia with no detectable vasospasm were tested again with ACh following pretreatment with intracoronary saline (*n* = 5) or 4.5 mg fasudil (*n* = 13)	- After the second Ach testing, the saline group developed ischemia; however, only 2 out of 13 treated with fasudil developed evidence of ischemia (*P* < 0.01) - Better lactate extraction ratio observed in the fasudil group (*P* = 0.0125)

Non-pharmacological approaches have a crucial role in managing ANOCA and INOCA. Cardiac rehabilitation which includes exercise training is beneficial in patients with ANOCA. A systematic review (107) of eight studies including 218 participants with exertional chest pain and absence of obstructive CAD who underwent cardiac rehabilitation including exercise programs with variable intensities concluded that exercise training improved exercise capacity and quality of life in most studies. Moreover, a pilot study on 16 patients with ANOCA who underwent an aerobic high-intensity training program for 3 months showed increased coronary flow velocity reserve (CFVR) and flow-mediated vasodilation (FMD) with exercise (108).

The use of a coronary sinus reducer is another non-pharmacologic novel approach to managing non-obstructive CAD. The device is an hourglass-shaped balloon expandable stent, which is implanted percutaneously in the coronary sinus and works by increasing the pressure in the sinus relieving angina (109). In a randomized clinical trial (109), 104 patients with myocardial ischemia and Canadian Cardiovascular Society (CCS) Class III or IV angina underwent a reducer implant surgery or a sham procedure. Results showed two CSS classes improvement in 35% of the treatment group compared with 15% of the control group at 6 months. Moreover, the quality of life significantly improved in patients undergoing reducer implantation according to the Seattle Angina Questionnaires scores. This has a potential use for patients with microvascular dysfunction.

Another non-pharmacological approach to the treatment of angina is enhanced external counterpulsation (EECP) which is non-invasive. It works by placing external inflatable cuffs on the lower extremities to increase the blood flow to the heart during diastole, which is followed by deflation of the cuffs during systole. A study (110) on the effect of EECP on patients with ANOCA and refractory angina assessed weekly anginal episodes before and after EECP treatment in 101 participants. Post-EECP treatment, CCS angina class, 6 min walk test, Duke Activity Status Index (DASI), Seattle Angina Questionnaire 7 (SAQ7), and weekly anginal episodes improved significantly. Moreover, a systematic review of 18 studies demonstrated the efficacy of EECP in the treatment of patients with refractory angina irresponsive to therapy (111).

Clinical experience would suggest management of epicardial vasospasm is usually responsive to medical therapy with good symptomatic relief. CMD, however, by experience, is a challenging entity for good symptomatic relief and often requires a combination and titration of medications tailored to the individual for relief. Invasive testing allows for some insight to help guide the appropriate therapy. Furthermore, a notable limitation of the current therapeutic approaches is the lack of extensive clinical trials, which necessitates the need for further research to guide clinical practice.

## 7 Conclusion

The prevalence of ANOCA and/or INOCA is increasing due to increased recognition and diagnosis, with CMD and epicardial coronary vasospasms as the most common underlying pathologies. Several risk factors are involved in the development of ANOCA and/or INOCA, including myocardial bridging, which is not well investigated and has been thought of as a benign condition. The prevalence of ANOCA and/or INOCA is not well investigated in the middle east, and further studies are needed in the region. Functional assessment of the coronary circulation is required to detect INOCA and can be done non-invasively or invasively, the latter being more complete. Moreover, trans-radial access is preferred. An interaction between acetylcholine used in spasm provocation testing and vasodilators to prevent RAS might be a concern and should be further investigated. Using vasodilators with the shortest half-life for RAS management, such as intra-arterial nitroglycerin, is a reasonable approach to minimizing the occurrence of any interaction and might lead to more accurate outcomes. The long-term management of INOCA involves pharmacological and non-pharmacological therapies and should be tailored to different underlying pathologies to achieve maximum benefit.
